# Complete Genome Sequence of the Fish Pathogen *Flavobacterium columnare* Strain C#2

**DOI:** 10.1128/genomeA.00624-16

**Published:** 2016-06-23

**Authors:** Ryan P. Bartelme, Ryan J. Newton, Yongtao Zhu, Nan Li, Benjamin R. LaFrentz, Mark J. McBride

**Affiliations:** a School of Freshwater Sciences, University of Wisconsin—Milwaukee, Milwaukee, Wisconsin, USA; b Department of Biological Sciences, University of Wisconsin—Milwaukee, Milwaukee, Wisconsin, USA; c Institute of Hydrobiology, Chinese Academy of Sciences, Wuhan, Hubei Province, China; d United States Department of Agriculture-Agricultural Research Service (USDA-ARS), Aquatic Animal Health Research Unit, Auburn, Alabama, USA

## Abstract

*Flavobacterium columnare* is a Gram-negative bacterial pathogen that causes columnaris disease of freshwater fish. *Flavobacterium columnare* strain C#2 was isolated from a diseased warm-water fish and is typed as genomovar II. The genome consists of a single 3.33-Mb circular chromosome with 2,689 predicted coding genes.

## GENOME ANNOUNCEMENT

*Flavobacterium columnare* is an important pathogen of freshwater fish (1–3). Although large die-offs among wild and farmed fish are caused by *F. columnare*, little is known regarding its virulence mechanisms or its ecology (4). Strains of *F. columnare* are classified into genomovars based on restriction digestion of the 16S rRNA gene and the 16S-23S rRNA internal transcribed spacer (2, 3), and *F. columnare* strain C#2 (originally referred to as *F. columnare* strain #2 [1, 5]) is a genomovar II strain. The genome sequence of a genomovar I strain was previously reported (6). *F. columnare* strain C#2 was selected for genome sequencing because it is a virulent genomovar II strain and is amenable to genetic manipulations (5).

A single colony of strain C#2 was grown overnight in 10 ml of modified Shieh medium (7) at 25°C with shaking. Genomic DNA (gDNA) was extracted from 10 ml of culture using the Qiagen DNeasy blood and tissue kit (Qiagen, Hilden, Germany). AMPure bead size-selected 20-kb libraries were constructed according to the Pacific Biosciences RSII protocol. Two PacBio single-molecule real-time (SMRT) cells were loaded with 0.01 and 0.015 nM concentrations of library with Pacific Biosciences sequencing reagent 4.0, C4 chemistry, and P6 version 2 polymerase. Sequencing produced 66,158 reads, with an *N*_50_ read length of 44,216 bp and mean read length of 20,496 bp, representing 296.7× coverage of the genome. Genome assembly was done using the PacBio PBcR HGAP 2.3.0 pipeline, with default settings (8). The assembly produced two contigs of 3,330,796 and 8,000 bases. The shorter 8-kb contig was eliminated, as the confidence scoring was too low for inclusion in the assembly (Quality score, mapQV < 10). Gepard comparison (9) of the *F. columnare* strain C#2 genome to that of *F. columnare* 94-081 (also genomovar II; accession no. CP013992), revealed three large syntenic regions split by a single chromosomal inversion and rearrangement. Similar chromosomal changes have been observed in other members of the genus *Flavobacterium* (10). The polished assembly was trimmed using the check_circularity.pl script from SPRAI (11), and the resulting single circular contig was annotated using the Prokaryotic Genome Annotation Pipeline through NCBI (12).

The C#2 genome has a G+C content of 30.97%, 13 rRNA operons, 93 tRNAs, and 2 clustered regularly interspaced short palindromic repeat (CRIPSR) arrays, and 93.4% of the open reading frames (ORFs) correspond to predicted coding genes. The genome was analyzed for secretion systems and potential secreted virulence factors (13, 14). *F. columnare* strain C#2 contains the core genes of the *Bacteroidetes*-specific type IX secretion system (15). Genes encoding potential secreted virulence factors, such as chondroitinases, proteases, and adhesins, were also identified. *F. columnare* strain C#2 exhibits gliding motility, which is potentially important in *columnaris* pathology, and the annotated genome contains all of the genes known to be required for this process (16, 17). The availability of a complete genome and the genetic manipulability of *F. columnare* strain C#2 enable further work toward understanding *F. columnare* virulence factors and the development of avirulent vaccine strains to prevent outbreaks of columnaris disease in aquaculture settings.

### Nucleotide sequence accession number.

The genome sequence of *Flavobacterium columnare* strain C#2 has been deposited in GenBank with the accession no. CP015107. This paper describes the first version of the genome deposited.
